# Where are aphasia theory and management “headed”?

**DOI:** 10.12688/f1000research.11122.1

**Published:** 2017-07-03

**Authors:** Donna C. Tippett, Argye E. Hillis

**Affiliations:** 1 Department of Neurology, Johns Hopkins University School of Medicine, Phipps 446, 600 N. Wolfe Street, Baltimore, MD, 21287, USA; 2 Department of Otolaryngology—Head and Neck Surgery, Johns Hopkins University School of Medicine, Baltimore, MD, USA; 3 Department of Physical Medicine and Rehabilitation, Johns Hopkins University School of Medicine, Baltimore, MD, USA; 4 Department of Cognitive Science, Johns Hopkins University, Baltimore, MD, USA

**Keywords:** aphasia, language processes, neuroimaging, neuromodulation, stroke recovery, rehabilitation

## Abstract

The sequelae of post-stroke aphasia are considerable, necessitating an understanding of the functional neuroanatomy of language, cognitive processes underlying various language tasks, and the mechanisms of recovery after stroke. This knowledge is vital in providing optimal care of individuals with aphasia and counseling to their families and caregivers. The standard of care in the rehabilitation of aphasia dictates that treatment be evidence-based and person-centered. Promising techniques, such as cortical stimulation as an adjunct to behavioral therapy, are just beginning to be explored. These topics are discussed in this review.

## Introduction

Communication through language is hampered by aphasia, an acquired disorder of language characterized by impairments in auditory comprehension, verbal expression, reading comprehension, and written expression ^1^. The most common cause of aphasia is a stroke involving the lateral aspects of the left cerebral hemisphere (for example, a left middle cerebral artery (MCA) infarct) ^2, 3^. The American Heart Association estimates that more than 795,000 strokes per year occur in the US ^4^. Aphasia is present in 15 ^5^ to 33% ^6^ of individuals with acute stroke. In addition, frequency of aphasia increases with advancing age, from 15% (95% confidence interval (CI) 5 to 26%) in patients younger than 65 years of age to 43% (95% CI 30 to 56%) among those at least 85 years of age ^6^. Post-stroke aphasia may be considered “a social condition” ^7^ that has considerable impact on functional recovery and societal costs. Costs for stroke-related health care exceeded $25 billion in 2007 ^4^. Reintegration into school, work, and family life may be precluded given human dependence on the spoken word, and social isolation is an all-too-common consequence of aphasia ^8^. Aphasia, in general ^9^, and specific language deficits ^10^ can necessitate discharge to more restrictive environments post-hospitalization so that accommodations can be provided to compensate for these impairments. Furthermore, post-stroke language impairments are troubling to patients and their caregivers. Difficulty with spelling and writing was the single most frequently reported important/moderate consequence of left hemisphere stroke by stroke survivors and their caregivers ^11^. Therapy is beneficial for language recovery; however, recovery can be variable and progress can be protracted, especially after large left hemisphere strokes ^12^.

In this review, we discuss theoretical models of the neural substrates of language and cognitive processes underlying aphasia that contribute to new models of neurobiological organization of language. Mechanisms of recovery of cognitive and language processes after stroke are reviewed along with current concepts of aphasia rehabilitation, including the promising role of cortical stimulation as an adjunct to behavioral therapy.

## Contemporary paradigms of neural substrates of language

Language is lateralized to the left hemisphere in approximately 96% of right-handed individuals and 70% of left-handed individuals ^13^, and so aphasia, a language disorder, results primarily though not exclusively from damage to the left hemisphere of the brain. Beginning in the 1980s, advances in neuroimaging, including positron emission tomography, functional magnetic resonance imaging (MRI), and magnetoencephalography, expanded understanding of the functional neuroanatomy of language. These safe, non-invasive imaging technologies revealed that language areas of the brain extended beyond Broca’s area and Wernicke’s area. Researchers learned that areas in both hemispheres of the brain are activated specifically during language tasks, although the left hemisphere shows more activation in the majority of neurologically normal adults ^14– 17^, and that more distant areas of the cortex, such as inferior and anterior temporal cortex ^8^, the basal ganglia and thalamus ^18^, and cerebellum ^19, 20^, are also activated during language tasks.

## Aphasia as disrupted cognitive/language processes

In addition to a new understanding of the neural complexity of language, there is increasing insight regarding the complexity of language tasks. Traditionally, aphasia has been classified according to classic vascular syndromes (that is, Broca’s aphasia, Wernicke’s aphasia, conduction aphasia, transcortical motor aphasia, transcortical sensory aphasia, mixed transcortical aphasia, anomic aphasia, and global aphasia) ^1^. Each vascular aphasia syndrome is defined by a collection of frequently co-occurring impairments that depend on an area of brain, supplied by a particular blood vessel (for example, the superior division of the left MCA supplying brain regions resulting in Broca’s aphasia with non-fluent, halting verbal output and the inferior division of the left MCA supplying brain regions resulting in Wernicke’s aphasia with fluent, verbose, low-content verbal output). Contemporary approaches characterize aphasia by disruption of specific cognitive processes. For example, access to semantic and lexical representations is needed to accomplish even a basic task, such as naming an object ^21– 23^. Cognitive representations are distributed across regions of the brain, and activation of these various areas is needed to evoke semantic representations. For example, the semantic representation of a horse includes features of how it moves (middle temporal visual area and middle superior temporal area), what it eats, and how humans use it. This approach to characterizing aphasia by disrupted cognitive/language operations is important for developing new theories of how language is represented and processed ^24^.

One such theory is the dual stream model, an innovative concept proposed by Hickok and Poeppel ^25– 27^, which includes a ventral stream for mapping sound onto meaning and a dorsal stream for mapping sound onto motoric productions and articulation. The ventral stream is a sound-meaning interface responsible for processing speech signals for comprehension. The ventral stream projects ventro-laterally and involves cortex in the superior temporal sulcus and the posterior inferior temporal lobe. In the dorsal stream, acoustic speech signals are translated into articulatory representations, essential for speech development and production, involving auditory-motor integration. The dorsal stream projects dorso-posteriorly toward the parietal lobe and ultimately to frontal regions. The dual streams are also thought to be bi-directional; the ventral stream mediates the relationship between sound and meaning for perception and production, and the dorsal system can also map motor speech representations onto auditory speech representations ^26, 27^. Although some aspects of this model are controversial and underspecified, current research is being carried out to refine the model and to determine the extent that it can provide a framework for rehabilitation.

The dual stream model is compatible with traditional aphasia classification ^28^. Superimposition of a map of the cerebrovascular territories onto Hickok and Poeppel’s neuroanatomical model reveals that the dorsal stream is supplied by the superior division of the left MCA and that the ventral stream is supplied largely by the inferior division of the left MCA. Individuals with the vascular syndrome of Broca’s aphasia present with non-fluent, telegraphic, poorly articulated verbal output that can be attributed to disruption of the dorsal stream: the articulatory network or sensorimotor interface. Those with the vascular syndrome of Wernicke’s aphasia have fluent, effortless, but relatively meaningless, spontaneous speech and repetition and have impaired comprehension at the word, sentence, and discourse levels that can be attributed to the lexical interface or combinatorial network (or both) to map sound onto meaning ^29^.

The concept of networks of brain regions is supported in a study of the controversial role of the anterior temporal lobe in cognition and language. Several studies recently concluded that the temporal pole is the “hub” of semantic processing, that it connects many other regions essential for semantics. However, Tsapkini, Frangakis, and Hillis ^30^ found no difference between patients with and without acute left temporal pole infarcts on auditory word comprehension and object-naming tasks. This finding suggests that damage to the left temporal pole is not sufficient to cause significant semantic deficits; instead, the temporal pole is likely part of a network responsible for comprehension and naming of objects. Similarly, other language skills, such as comprehension of yes/no questions and verbal working memory, are associated with multiple brain regions and their connections ^31, 32^.

## Mechanisms of recovery

Mechanisms of recovery after stroke include restoration of blood flow, recovery from diaschisis (that is, language impairment that is caused by loss of input because of a remote lesion functionally connected to the cortical areas responsible for that language ability), and reorganization of structure-function relationships in the brain associated with neuroplasticity (that is, the adaptive ability of the brain to reorganize and modify tissue functions in the setting of pathology). Medical, surgical, and pharmacological interventions are employed to augment recovery. Acute stroke interventions, such as medically induced blood pressure elevation, thrombolysis, embolectomy, and stenting, restore blood flow to ischemic tissue that is receiving enough blood to survive but not enough to function (“ischemic penumbra”). These interventions can augment aphasia recovery by allowing recovery of tissue function before there is permanent damage to the entire affected area ^33– 37^. Pharmacological interventions for aphasia are mainly designed to strengthen networks subserving language and language-related cognitive functions such as attention and memory ^38^. The theoretical rationale for pharmacological intervention in aphasia is based on the notion that re-establishing the activity of specific neurotransmitters (typically noradrenergic, dopaminergic, cholinergic, and glutamatergic neurotransmitter systems) in dysfunctional, but not irretrievably damaged, brain regions may strengthen neural activity in networks mediating attention, word learning, and memory ^39, 40^.

Recovery from diaschisis was described in an individual who showed near absence of left hemisphere activation during a word-generation task at baseline despite no hypoperfusion or structural disconnection. At 8 weeks post-stroke, there was activation of the left hemisphere ^37^. Recovery from diaschisis was also reported in a case series of 10 individuals with isolated left thalamic lesions. Five of the 10 individuals had aphasia; one had cortical hypoperfusion. This suggested that naming and auditory comprehension deficits were not attributable to left cortical hypoperfusion, but instead were caused by dysfunction of the thalamic-cortical system via diaschisis ^41^.

In chronic stroke, recovery may occur via reorganization, such that intact areas of the brain assume the function of a damaged area. This type of recovery requires time and thus is seen in chronic rather than acute stroke ^25^. For example, Broca’s aphasia is associated with stroke involving the posterior, inferior frontal gyrus (Brodmann areas 44 and 45) ^29^. This association is more consistent in acute than chronic stroke, indicating that structure-function relationships are reorganized over time ^42^. Activation of right hemisphere homologs of language areas and perilesional areas may compensate for damaged language areas of the brain ^43– 46^. Saur *et al.* ^47^ found that cortical activation changed over time in an individual who had good recovery of language function, with little activation in either hemisphere during an auditory sentence comprehension task in the acute post-stroke phase, predominately right hemisphere activation in the subacute phase, and a return to mainly left hemisphere activation in the chronic phase.

In addition, the impact of neuroimaging on the study of brain-behavior relationships and stroke recovery is substantial. The blood oxygen level-dependent (BOLD) signal on functional MRI shows areas where blood flow exceeds oxygen extraction, which corresponds to activation of neurons. Saur *et al.* ^48^ showed that BOLD activity in the right inferior frontal cortex, along with clinical data, improves prediction of language recovery at 6 months post-stroke. Diffusion tensor imaging (DTI) reveals white matter tracts by identifying areas where water molecules flow in the same direction. Using DTI imaging, Forkel *et al.* ^49^ found that the volume of the long segment of the arcuate fasciculus in the right hemisphere (contralateral to the lesion) is an important predictor of recovery of language after stroke.

## Treatment: behavioral approaches and neuromodulation

Aphasia treatment is progressively more informed by advances in understanding of the neurobiology of recovery and learning. Principles of neuroplasticity support early and intense therapy. Plasticity studies reveal the functional importance of the “use it or lose it” principle and indicate that beneficial behavioral and neural changes can be effected through intense and repetitive practice ^50^. Findings of early investigations of aphasia therapy emphasize that intense treatment for short periods is more effective than a similar number of therapy sessions over longer periods ^51^. More recently, the role of intensity of therapy, rather than therapeutic approach, is shown through the similar treatment outcomes achieved by stroke survivors whether they received conventional versus constraint-induced therapy ^52^. The rationale for early intervention in aphasia is based on these neuroplasticity principles such that therapy capitalizes on spontaneous recovery in the immediate post-stroke period ^53^. In chronic stroke, constraint-induced therapy is thought to stimulate cortical reorganization by encouraging verbal (versus non-verbal) communication ^54, 55^.

Application of principles governing brain organization and reorganization may contribute to the development of more meaningful therapy goals. For example, practice on a confrontation naming task may facilitate the ability to convey communicative intentions to listeners as a result of the adaptive property of the brain. Treatment goals may also be reframed on the basis of the dual stream model of language organization. For example, for those with Broca’s aphasia, therapy may be directed at translating sound to motor speech productions to produce simple sentences, whereas those with Wernicke’s aphasia may be directed to processing speech for comprehension or meaning in sentences ^56^. Further investigation is warranted regarding how the segregation of language functions described by this model suggests particular approaches that promote “use” most effectively. One suggestion is that ventral stream could be accessed by instructing patients to process the meaning of a target word during a repetition task in the treatment of conduction aphasia ^57^.

Current practice standards dictate that therapy must be evidence-based and person-centered. Evidence-based practice refers to an approach in which current, high-quality research evidence is integrated with practitioner expertise and client preferences and values ^58^. The hierarchy and generalizability of evidence are evaluated ^59, 60^ and an individual’s life circumstances, preferences, coping mechanisms, and concomitant medical, sensory, behavioral, and psychological issues are considered when making treatment decisions. Clinicians combine multiple, available studies of sufficiently good design, expert consensus, and clinical knowledge of anatomy and physiology to make reasonable judgments about the appropriateness and effectiveness of a specific treatment technique ^61^. A growing literature documents the evidence base for speech-language pathology treatment of aphasia (for example, ^62– 66^).

Person-centered practice “involves valuing the individual needs and rights of patients, understanding patients’ illness and health care experiences, and embracing them within effective relationships which enable patients to participate in clinical reasoning” ( 67, p. 68). The life participation approach to aphasia is an example of a patient-centered therapy paradigm ^68^, although clinicians can tailor specific therapy tasks to meet individuals’ unique needs ^69^. Specific tasks can also be adapted to conform to a patient-centered approach. For example, the Activity Card Sort ^70^ can be tailored to elicit information from individuals with aphasia about their level of engagement in meaningful activities as well as hindrances to participation, allowing clinicians to obtain qualitative information about interests, level of involvement, and priorities which then could be used to shape the direction of therapy. Social models of therapy encompass the authentic involvement of users (patients), creation of engaging experiences, user control, and accountability ^71^. This practice is consistent with the conceptual framework for contemporary models of health care of the International Classification of Functioning, Disability, and Health of the World Health Organization ^72^.

Non-invasive brain stimulation offers a potentially important adjunctive approach to behavioral therapy, such as transcranial magnetic stimulation (TMS) and transcranial direct current stimulation (tDCS). Cotelli *et al.* ^73^ hypothesized that TMS and tDCS can facilitate neuroplasticity through the reactivation of canonical networks and recruitment of compensatory networks and perilesional areas. TMS has been used to treat naming in individuals with non-fluent aphasia ^74, 75^. Two recent meta-analyses explored the utility of repetitive transcranial magnetic stimulation. Li *et al.* ^76^ reported on four articles in which 132 patients received inhibitory TMS which facilitated improvements in naming more so than repetition or comprehension. In the second meta-analysis, Ren *et al.* ^77^ demonstrated that inhibitory TMS to the right inferior frontal gyrus of patients with subacute and chronic aphasia enhanced language recovery, as measured by aphasia severity, expressive language, and receptive language.

tDCS promotes neuroplasticity by modulation of spontaneous cortical activity in the brain. tDCS involves application of low-amplitude direct current to the scalp via two surface electrodes that modulate the excitability of cortical neurons without directly inducing neuronal action potentials ^78^. The effects of the stimulation depend on the polarity of the current flow, and brain excitability is usually increased by anodal tDCS and decreased by cathodal tDCS ^79^. Some studies have examined the effect of anodal or excitatory tDCS applied to the lesioned left hemisphere to improve language recovery via enhancement of neuronal activity in the perilesional cortical area ^80– 83^. Other studies have examined the effect of cathodal or inhibitory tDCS applied to the contralateral hemisphere to decrease activity in right hemisphere to improve language function (for example, ^84– 86^). The promise of these methods relies on a full understanding of the anatomy of the neural networks underlying language and variables that influence potential timing and extent of structure-function reorganization.

## Conclusions

In this review, the question “Where are aphasia theory and management ‘headed’?” is addressed with respect to new insights regarding the neurologic foundation of language, characterization of aphasia in the context of cognitive processes, and advances in treatment, including medical, surgical, pharmaceutical, behavioral, and neuromodulatory options. Challenges abound. These include how to expand speech-language pathology treatment to address the disrupted cognitive processes of aphasia and how to modify and supplement behavioral modes of treatment to optimize outcomes. Evidence of effectiveness of methods to deliver cortical stimulation is preliminary but promising; further research is indicated to establish the mechanism associated with language recovery after these novel treatments. Addressing these issues requires a sound clinical knowledge base, persistence, and creativity.
